# Multicenter study of the evolution of different types of avulsion over the 12 months after delivery

**DOI:** 10.1002/ijgo.14208

**Published:** 2022-04-27

**Authors:** José Antonio García‐Mejido, Enrique González‐Diaz, Ismael Ortega, Alicia Martín‐Martinez, Ana Fernández‐Palacín, José Antonio Sainz‐Bueno

**Affiliations:** ^1^ Department of Obstetrics and Gynecology Valme University Hospital Seville Spain; ^2^ Department of Obstetrics and Gynecology University of Seville Seville Spain; ^3^ Department of Obstetrics and Gynecology Complejo Asistencial Universitario de Leon (CAULE) León Spain; ^4^ Department of Obstetrics and Gynecology Complejo Asistencial Universitario de Gran Canarias Gran Canarias Spain; ^5^ Biostatistics Unit, Department of Preventive Medicine and Public Health University of Seville Seville Spain

**Keywords:** cervical elongation, pelvic floor, pelvic organ prolapse, three‐dimensional transperineal ultrasound, uterine prolapse

## Abstract

**Objective:**

To perform a multicenter study of muscle recovery in levator ani muscle (LAM) avulsion during the first 12 months postpartum according to the type of LAM avulsion suffered.

**Methods:**

This was a multicenter prospective observational study including 242 primiparas. Transperineal ultrasound was performed at 6 months and 12 months after delivery. Type I LAM avulsion was present when most of the lateral fibers of the pubovisceral muscle were observed at the muscle's insertion at the pubic level. Type II LAM avulsion was defined as complete detachment of the pubovisceral muscle from its insertion at the pubic level.

**Results:**

Among the 56 patients who completed the study (with ultrasound at 6 and 12 months after delivery), 76 avulsions (10 cases of bilateral avulsion) were identified at 6 months after delivery, and the total number of avulsions had decreased to 58 at 12 months after delivery (*P* < 0.001; 95% confidence interval [CI] 13.9%–33.5%). This decrease was due to the disappearance of 69.2% of the cases of Type I LAM avulsions (*P* < 0.001; 95% CI: 50.2%–88.2%). However, the number of Type II LAM avulsions remained constant at 6 months and 12 months after delivery.

**Conclusion:**

The spontaneous resolution of LAM avulsion during the first 12 months postpartum occurs in cases of Type I LAM avulsion but is not observed in Type II LAM avulsion.

## 1 INTRODUCTION

Levator ani muscle (LAM) avulsion is defined as detachment of the fibers of the LAM from the muscle's insertion at the level of the inferior pubic branch.^1^ Its occurrence is related to different pathologies of the pelvic floor, such as pelvic organ prolapse,^2^, ^3^ voiding dysfunction,^4^ and relapse after prolapse correction surgery.^5^ However, its association with stress urinary incontinence is not as evident.^6^

LAM avulsion is intimately related to vaginal delivery,^7^ occurring when the vertex of the fetal head is at station +3 to +4.^8^, ^9^ Although the reported rates of LAM avulsion range from 12% to 36% of vaginal deliveries,^1^, ^10^, ^11^ it has been determined that these values decrease throughout the first year postpartum.^12^, ^13^ This fact has given rise to two theories that attempt to justify the disappearance of LAM avulsion over time. The first theory postulates that an excessively early ultrasound examination after delivery can favor diagnostic errors.^12^, ^14^, ^15^ The second theory focuses on the possible natural healing^12^, ^14^, ^16^, ^17^ of LAM avulsion over time. However, neither of these two assumptions can justify the observed decrease in avulsion rates over time. Anatomy and magnetic resonance imaging have confirmed that there are two types of LAM avulsion^18^; of these, spontaneous resolution of Type I LAM avulsion occurs between the ultrasound examinations performed at 6 months and 12 months after delivery.^19^

The main problem that must be verified is that while some LAM avulsions disappear during the first 12 months after delivery, there are few studies that analyze the differences in the rates of LAM avulsion throughout the first postpartum year,^12^, ^13^, ^19^ and only one study has analyzed LAM avulsion type, determining that it is not possible to recover from LAM avulsion after 12 months postpartum. Consequently, the objective of our work is to conduct a multicenter study to determine whether muscle recovery of LAM avulsion occurs during the first 12 months postpartum according to the type of LAM avulsion.

## 2 MATERIALS AND METHODS

A multicenter prospective observational study was designed with 242 patients who were consecutively recruited after delivery and before hospital discharge between February 1, 2018 and April 1, 2019. The hospitals included in the study were the Valme University Hospital of Seville (Spain), the University Health Care Complex of Gran Canaria (Spain), and the University Health Care Complex of León (Spain).

The inclusion criteria for the study were nulliparity, full‐term gestation, cephalic presentation, vaginal delivery, no previous pathology of the pelvic floor, and having previously provided written informed consent. Patients who did not present LAM avulsion at 6 months postpartum were excluded from the study. The patients were recruited verbally, after delivery, by each examiner responsible for each center (JAGM, EGD, and IO). The signed informed consent forms were obtained by the same people in each center, per local ethical standards. Patient data and ultrasound volumes were coded and stored by those responsible (JAGM, EGD, and IO) to ensure the anonymity of the included patients.

The general characteristics of interest were maternal age; gestational age; induction of labor; epidural analgesia; duration of the epidural; duration of the second stage of labor; type of vaginal delivery (normal, vacuum or forceps); the presence of episiotomy (restrictive^20^ or nonsystematic episiotomy) and high‐grade perineal tears, according to the Sultan classification of perineal tears^20^; and fetal weight and head circumference.

**FIGURE 1:**
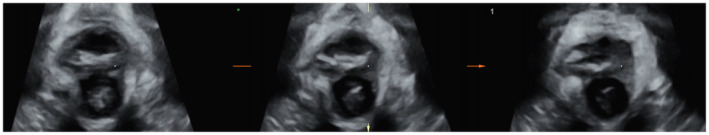
Right avulsion with Type I levator ani muscle avulsion where most lateral fibers of the pubovisceral muscle were observed at its insertion at the pubic level

**FIGURE 2:**
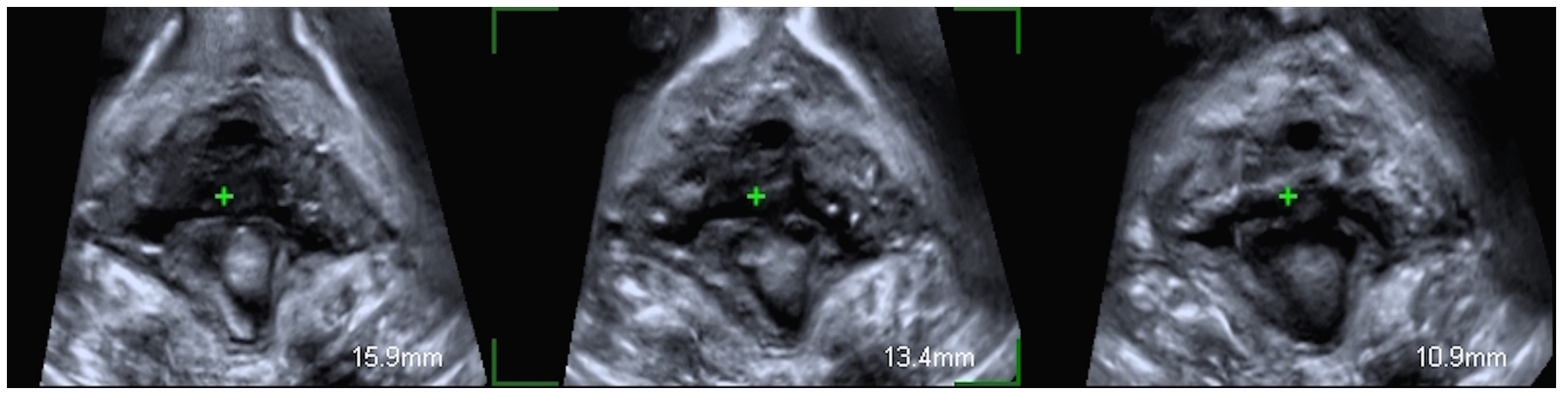
Bilateral avulsion with Type II levator ani muscle avulsion, defined as the complete detachment of the pubovisceral muscle from its insertion at the pubic level

Transperineal ultrasound was performed 6 months and 12 months after delivery by expert examiners at each hospital (JAGM, EGD, and IO). The examiners were blinded to the obstetrical data related to the delivery. The ultrasound machines used were a Toshiba 500 Aplio® (Toshiba Medical Systems Corp.) with a PVT‐675 MV three‐dimensional (3D) abdominal probe and a Voluson E8 (GE Health Care) ultrasound system with an 8‐ to 4‐MHz volume transducer covered with a sterile glove. The 3D/4D ultrasound images were acquired from the mean sagittal plane images, as described above.^21^ The ultrasound volumes were obtained by positioning patients with empty bladders in the dorsal lithotomy position.^21^ The ultrasound volumes of the pelvic floor were performed in 4D under three conditions for each exploration: at rest, at maximum contraction, and with the Valsalva maneuver (for at least 6 s).^22^ The levator hiatus area was studied in the plane of minimal hiatal dimension. Avulsion was defined under maximum contraction conditions in the multislice mode.^23^, ^24^ The diagnosis of complete avulsion was made when there was abnormal insertion of the LAM in the three central slices. When abnormality of the insertion was unclear, a levator‐urethra gap greater than 2.5 cm was used to define an abnormal insertion.^25^ Type I LAM avulsion was present when the pubovisceral muscle insertion was partially attached at the pubic level (the arch of the elevator remained intact, as indicated with a white arrow in the left avulsion presented in Figure 1).^19^ Type II LAM avulsion was defined as the complete detachment of the pubovisceral muscle from its insertion at the pubic level (see the right avulsions in Figure 2).^19^

Thirty‐five women were needed to detect a difference of 35% in the paired percentages of avulsion between 12 months (70%) and 6 months (35%), with an error of 5% and a 1–β power of 80% and considering the bilateral nature of the test.

For continuous variables, the normality of the data was assessed (using Shapiro–Wilk test); analysis of variance for independent samples or a nonparametric Kruskal‐Wallis test was applied, followed by a multiple comparison test if the variable was significant in the previous test. For the analysis of categorical variables, either contingency tables plus χ^2^ tests or non‐asymptotic Monte Carlo methods plus Fisher exact tests were performed. Paired categorical variables were analyzed using McNemar test. A value of *P* less than 0.05 was considered statistically significant. The statistical analysis was performed using the SPSS program version 26 (IBM).

The study was approved by the Biomedical Ethics Committee of the Junta de Andalucía (1459‐N‐18).

## 3 RESULTS

Of the 242 patients who were recruited, 59 presented at least one LAM avulsion at 6 months after delivery (first ultrasound), and of these, 56 completed the study by undergoing another ultrasound at 12 months after delivery. Three patients with avulsion did not receive the second ultrasound, and 10 had no bilateral avulsions identified. The general and obstetric characteristics of the 56 patients who completed the study are presented in Table 1. The levator hiatus area at 6 months after delivery was 17.82 ± 4.36 cm^2^ (mean ± standard deviation) at rest, 23.95 ± 6.03 cm^2^ with Valsalva maneuver, and 16.96 ± 4.83 cm^2^ with contraction; at 12 months after delivery, it was 16.27 ± 3.51 cm^2^ at rest, 21.99 ± 6.61 cm^2^ with Valsalva maneuver, and 13.76 ± 3.18 cm^2^ with contraction (Table 2).

**TABLE 1:** General obstetric characteristics (*n* = 56)^a^

Characteristic		95% CI
Maternal age, year	31.86 ± 4.75	30.59–33.13
Weight, kg	73.69 ± 10.84	70.73–76.65
Height, m	1.63 ± 0.06	1.62–1.65
BMI	27.65 ± 3.74	26.63–28.67
Gestational age	39.77 ± 1.25	39.43–40.10
Induced labor	16/56 (28.6%)	18.0%–41.3%
Epidural	52/56 (92.9%)	83.9%–97.5%
Epidural period, min	344.23 ± 274.28	259.82–428.64
Second stage of labor, min	126.45 ± 78.95	105.30–147.59
Delivery		
Normal	4/56 (7.1%)	2.5%–16.1%
Vaccum	31/56 (55.4%)	42.3% –67.5%
Forceps	21/56 (37.5%)	25.7%–50.5%
Episiotomy	51/56 (91.1%)	81.5%–96.5%
Perineal tear	21/53 (39.6%)	27.3%–53.1%
Grade I	7/21 (33.3%)	16.3%–54.6%
Grade II	8/21 (38.1%)	19.9%–59.3%
Grade III	6/21 (28.6%)	12.9%–49.7%
Newborn weight, g	3448.48 ± 421.56	3335.59–3561.38
Fetal head circumference, cm	34.89 ± 1.29	34.51–53.28

Abbreviations: BMI, body mass index (calculated as weight in kilograms divided by the square of height in meters); CI, confidence interval.

^a^ Values are given as mean (standard deviation) or as number/total number (percentage), unless otherwise stated.

**TABLE 2:** Levator hiatus ultrasound measurements at 6 months and 1 year after delivery^a^

	Measurements		*P* value	95% CI
	6 months after delivery (*n* = 56)	12 months after delivery (*n* = 56)		
Levator hiatus area, cm^2^				
Rest	17.82 ± 4.36	16.27 ± 3.51	0.005	0.49–2.60
Valsalva	23.95 ± 6.03	21.99 ± 6.61	0.006	0.59–3.33
Contraction	16.96 ± 4.83	13.76 ± 3.18	<0.001	2.03–4.39

Abbreviation: CI, confidence interval.

^a^ Values are given as mean ± standard deviation, unless otherwise stated.

The total number of avulsions decreased from 76 to 58 between 6 months after delivery and 12 months after delivery (*P* < 0.001; 95% confidence interval [CI] 13.9%–33.5%). This decrease was due to the disappearance of 69.2% of cases of Type I LAM avulsion (*P* < 0.001; 95% CI: 50.2%–88.2%), including 64.3% of the left avulsions (*P* < 0.001; 95% CI: 35.6%–93.0%) and 75% of the right avulsions (*P* < 0.001; 95% CI: 46.3%–100%). However, the number of Type II LAM avulsions, both left and right, remained constant between 6 months and 12 months after delivery (Table 3).

**TABLE 3:** Levator ani muscle avulsions at 6 months and 12 months after delivery^a^

	Total number of avulsions (*n* = 76)	LAM avulsion Type I (*n* = 26)	Left (*n* = 14)	Right (*n* = 12)	LAM avulsion Type II (*n* = 50)	Left (*n* = 31)	Right (*n* = 19)
6 months after delivery	76/76 (100%)	26/26 (100%)	14/14 (100%)	12/12 (100%)	50/50 (100%)	31/31 (100%)	19/19 (100%)
12 months after delivery	58/76 (76.3%)	8/26 (30.8%)	5/14 (35.7%)	3/12 (25%)	50/50 (100%)	31/31 (100%)	19/19 (100%)
*P* value	<0.001	<0.001	<0.001	<0.001	—	—	—
95% CI	13.9%–33.5%	50.2%–88.2%	35.6%–93.0%	46.3%–100%	—	—	—

Abbreviations: CI, confidence interval; LAM, levator ani muscle.

^a^ Values are given as number/total number (percentage), unless otherwise stated.

## 4 DISCUSSION

We observed a decrease in the diagnosis of Type I LAM avulsions between 6 months and 12 months after delivery. At 6 months postpartum, there were 14 left Type I LAM avulsions and 12 right Type I LAM avulsions; at 12 months, there were five left Type I LAM avulsions (decrease of 64.3%, *P* < 0.001; 95% CI: 35.6%–93.0%) and three right Type I LAM avulsions (decrease of 75%, *P* < 0.001; 95% CI: 46.3%–100%). However, for the Type II LAM avulsions, there was no change in the number of diagnoses between 6 months and 12 months after delivery. This is consistent with previously published findings showing that Type II LAM avulsion remained constant over time.^19^ However, this previous study reported that all Type I LAM avulsions disappeared between 6 months and 12 months after delivery,^19^ but we observed that not all Type I LAM avulsions disappeared during that period.

The reason for this disappearance of LAM avulsion is not clear. Some authors propose that it is due to a process of spontaneous healing^12^ in the first 6 months after delivery.^14^ In fact, the repair process could be similar to that which occurs in other muscle injuries, producing degeneration, inflammation, regeneration, and finally fibrosis of the injured muscle. The disappearance of LAM avulsion throughout the postpartum period has been described by other authors. Branham et al.^26^ detected a recovery of avulsion between 6 weeks and 6 months after delivery in primiparous women, and other authors have described variable LAM avulsion rates of between 20.8% and 62%^12^, ^13^ due to the disappearance of avulsion 1 year after delivery. Valsky et al.^17^ found that 19% of avulsions observed on 3D transperineal ultrasound disappeared during follow up. Shek et al.^16^ observed that of 12 cases of avulsion established at 3–6 months after delivery, three avulsions had disappeared at 1 year. When we analyzed the published images in the articles that refer to the recovery of the injury,^12^, ^13^, ^16^, ^17^ we observed that the cases of avulsion recovery corresponded to Type I LAM avulsions. These data show that Type I LAM avulsion is a lesion that can recover over time. Our ultrasound findings are consistent with the previously described lesions in terms of anatomy and magnetic resonance imaging findings^18^ and support the possibility that the injury recovers over time. Kim et al.^18^ defined Type I LAM avulsion as limited to individual portions of the pubovisceral muscle, with some parts of the muscle missing while the arch of the elevator remains intact. The maintenance of this arch of the intact elevator could favor muscle recovery at that level. On the other hand, Kim et al.^18^ defined Type II injury as involving the detachment of the catenary‐like levator arch from the pubic bone as a result of excessive tension in this region during vaginal birth. This Type II injury involves a loss of the normal architecture of the pelvic sidewall due to loss of this critical attachment, making spontaneous muscle recovery difficult.

Other authors have postulated that the disappearance of avulsion could be a result of diagnostic errors related to an excessively early first assessment.^12^, ^14^, ^15^ A recent systematic review advised practitioners to avoid performing imaging tests during the early postpartum period owing to the risk of overdiagnosis; consequently, it seems reasonable to postpone a final diagnosis until 6 months after delivery or until more than 12 months after forceps‐assisted delivery.^27^ Furthermore, it has been suggested that the current criteria for the diagnosis of LAM avulsion may not be appropriate after the first delivery,^15^ because the diagnosis may depend on the clinician. Therefore, we performed a multicenter study with the objective of verifying that LAM avulsion occurs in a similar way regardless of the center, examiner, or type of ultrasound used.

One of the strengths of our study is its multicenter design, which ensured that diagnosis was performed by different examiners and with different ultrasound equipment. In addition, the study examined an ultrasound diagnostic concept that is easy to apply and has been previously described in anatomical studies and by magnetic resonance imaging.^18^ However, we also detected areas for improvement, such as the need to continue studying the evolution of Type I LAM avulsion for more than 12 months after delivery to observe its change over time. In addition, new studies should include a greater number of patients with Type I LAM avulsion to obtain results focused on this type of lesion.

In conclusion, we observed that the resolution of LAM avulsion during the first 12 months postpartum occurs in cases of Type I LAM avulsion and is not observed in patients with Type II LAM avulsion.

## AUTHOR CONTRIBUTIONS

JAG‐M was responsible for the conception and design of the study. JAG‐M and JAS provided administrative support. JAG‐M, EG‐D, IO, and CB provided study materials or patients, and collected and assembled the data. JAG‐M, AF‐P, and JAS performed the data analysis and interpretation. All authors wrote the manuscript and gave final approval of the manuscript.

## CONFLICTS OF INTEREST

All authors have completed the ICMJE uniform disclosure form. The authors have no conflicts of interest to declare.

## Data Availability

Research data are not shared.
